# Editorial: What Next - The Cognition of Sequences

**DOI:** 10.3389/fpsyg.2017.02160

**Published:** 2017-12-13

**Authors:** Snehlata Jaswal

**Affiliations:** LM Thapar School of Management, Thapar University, Punjab, India

**Keywords:** working memory, sequences, cognition, sequential learning, sequential effects

Sequences are ubiquitous in our lives, yet mysterious and difficult to research because of many confounds. The research topic “What next: The Cognition of Sequences” aimed to understand sequencing behavior by gathering theoretical and empirical articles showcasing current views. Gratifyingly, contributions not only addressed the theoretical debates but also focused on memory for sequences in special populations such as the autistic and the dyslexic, hinting at possible applications in this area. The recurring theme in the contributions is that sequencing is a process within Working Memory rather than merely a perceptual entity.

Proposing a unified theoretical framework for cognitive sequencing, Savalia et al. bring together two diverse debates in the sequencing literature—the implicit vs. explicit nature of sequencing, and the goal directed vs. habit-oriented response systems. They propose that the brain implicitly (automatically) extracts regularities from the myriad, ever changing stimuli, but uses attention to organize them in a hierarchical way. Attention is also needed to organize sequences of responses/ actions to achieve a future goal, although when repeated often enough, these sequences acquire the force of habit with a concomitant release from the processes of attention. This theoretical framework serves for both humans and animals, and is perhaps best exemplified by skill acquisition.

In line with these thoughts, Rogers et al. provide empirical evidence that statistical learning of stimulus sequences is indeed implicit, being unaffected by reward contingencies. In their experiment, they found significant visual statistical learning effects, but no-, low-, or high-reward conditions did not cause any differences in the strength of learning. Thus, the amount of rewards did not affect statistical learning of sequences. They conclude that the system that detects links and regularities among stimuli, functions independently of the system that identifies reward contingencies.

Poth and Schneider explore how we remember objects from previous episodes. This could be because we remember visual features of objects or we remember the objects stored in VWM. Using a new paradigm combining letter report and probe recognition, they evaluated the dependence of episodic short term recognition on VWM. The first experiment showed that participants recognized probes more often if they had reported them earlier in a whole report. The second experiment required partial report of one letter, and probes were either for this letter, or those near it, or those far from it. Probe recognition was better for near than for far letters, indicating that episodic short term recognition is only possible for a limited number of simultaneously presented objects due to the encoding limitation of VWM. presentations.

De Lillo et al. provide evidence for VWM factors being more crucial than perceptual grouping in the retention of spatial sequences. They used variants of the Corsi task on touch screen monitors and in virtual reality to establish that serial spatial recall is least affected by path length. It is the structure or organization imposed on the stimuli which is the most important factor in the performance of the participants. Their experiments show that visual perceptual grouping factors are not necessary for the benefit of structure, and thus they conclude that encoding of structure happens at a post perceptual stage in VWM.

Manohar and Husain explored the retention of sequences of brief time intervals in the auditory modality, which were to be reproduced by the participants by holding down a key. Analogous to verbal and visuo-spatial working memory, they found an effect of set size as well as serial position effects. Attention and expectation were also significant factors. Performance was significantly worse when only one item (the lowest set size) was to be remembered, indicating the vulnerability of memory for single items. They conclude the mechanisms used to remember auditory time durations in Working Memory are similar to those used to remember verbal or visual stimuli.

Johnson et al. compared children and adults in their orientation/attention to temporal vs. spatial cues. In one experiment, location of a target was predicted by an arrow, while target onset was predicted by a short or long tone. Adults' showed a greater response latency in invalid trials as compared with valid trials in both spatial and temporal domains. However, children's (Mean age 11.4 years) responses were slowed only in the spatial domain, and were not affected in the temporal domain. In the second study, a series of sounds were presented in a rhythmic series. In this experiment, children were slowed significantly by invalid cues. Thus, sequential rather than single presentation, helped children orient attention in time.

Sequences, are however, not only important for perception of time. Deficits in sequencing are associated with other problems as well. Tsai et al. provide evidence of deficits in processing of simple cue-target sequences being associated with childhood obesity. They compared the performance of children with obesity and healthy weight controls using behavioral as well as ERP measures on a visuospatial attention task. The task used the Posner paradigm in which the children had to respond to a cue-target sequence correctly as well as quickly. Simultaneously, ERP activity was recorded. Children with obesity showed poorer behavioral performances (slower reaction times as well as deficits in attentional inhibition) and aberrant neural activity (e.g., smaller P3 amplitudes) when performing the task.

Majerus and Cowan review the evidence regarding verbal STM shortcomings in people with dyslexia. Their contention is that this STM impairment in dyslexics is primarily an inability to process serial order in working memory. This impairment is found for verbal as well as non-verbal (visuo-spatial) material. However, it is not reported by every individual who has dyslexia, nor is it specific to only dyslexics. Thus, it is not a cognitive marker of dyslexia. Nor is it clear whether and how far this impairment contributes causally to dyslexia. Finally, research is also needed to disentangle the mechanisms and effects of deficits in serial order processing and phonological processing.

Age differences in forward and backward recall are documented in an original research article by Brown. She compared young adults (18–40 years) and older adults (64–85 years) on a modified version of the Spatial Span subtest of the Wechsler Memory Scale. Spatial interference had the maximum effect as compared to visual interference and a control condition, indicating that the task was indeed assessing spatial memory. Further, using regression analyses within each age group, she provides evidence regarding age being a significant factor in backward spatial span performance, supporting other studies which show declines in a variety of working memory tasks with increasing age. Her study also shows backward span is more sensitive to aging than forward span, presumably because it relies more heavily on processing than forward span task.

Donolato et al. review the literature regarding differences in forward and backward order recall in the verbal and visuo-spatial domains of Working Memory. They begin with the proposition that order of presentation is crucial in verbal but nor visuo-spatial memory. This is particularly evident in differences in forward and backward recall, with performance being worse in backward than forward recall in verbal tasks, but not always so in visuo-spatial tasks. Nevertheless, their mini review shows that in individuals with weak visuospatial abilities, performance is worse for backward recall than for forward recall. This indicates the importance of individual differences in cognitive tasks in general and Working Memory tasks in particular.

The commentary by Dubrow and Davachi on an original fMRI research article by Jenkins and Ranganath (2016) regarding neural mechanisms underlying the memory for events is included in the research topic because it provides an insightful comparison of various mechanisms that extant literature suggests support memory for temporal order. The authors begin the review with the intuitive notion that the item memory strength gives a cue regarding which of two stimuli is the most recent one. They move on to context differentiation account of order memory. Then they discuss the theories of temporal representation based on absolute time and position at which an event occurred, and those which are based on relative time and position (associative chaining). Their paper also addresses how fMRI data can be used to test these competing viewpoints, suggesting future avenues of research.

To conclude, it seems sequences are constructed within Working Memory from the raw material provided by perception, sometimes implicitly, but more often explicitly. Further this process is important in intact cognitive processing, and aberrations are associated with behavioral/clinical problems which we are just beginning to explore. Future research is envisioned in two related avenues—determining the factors and processes in Working Memory which contribute to sequencing, and studying how to ameliorate problems of sequencing behavior through education and training in general, and treatment and rehabilitation efforts in clinical populations.

## Author contributions

The author confirms being the sole contributor of this work and approved it for publication.

### Conflict of interest statement

The author declares that the research was conducted in the absence of any commercial or financial relationships that could be construed as a potential conflict of interest.
